# Air Annealing Effect on Oxygen Vacancy Defects in Al-doped ZnO Films Grown by High-Speed Atmospheric Atomic Layer Deposition

**DOI:** 10.3390/molecules25215043

**Published:** 2020-10-30

**Authors:** Chia-Hsun Hsu, Xin-Peng Geng, Wan-Yu Wu, Ming-Jie Zhao, Xiao-Ying Zhang, Pao-Hsun Huang, Shui-Yang Lien

**Affiliations:** 1 School of Opto-electronic and Communication Engineering, Xiamen University of Technology, Xiamen 361024, China; chhsu@xmut.edu.cn (C.-H.H.); gexipe@163.com (X.-P.G.); 2015000077@xmut.edu.cn (M.-J.Z.); xyzhang@xmut.edu.cn (X.-Y.Z.); 2 Department of Materials Science and Engineering, Da-Yeh University, ChungHua 51591, Taiwan; wywu@mail.dyu.edu.tw; 3 School of Information Engineering, Jimei University, Xiamen 361021, China; ph.huang@jmu.edu.cn; 4 Fujian Key Laboratory of Optoelectronic Technology and Devices, Xiamen University of Technology, Xiamen 361024, China

**Keywords:** atomic layer deposition, aluminum-doped zinc oxide, annealing, oxygen vacancy

## Abstract

In this study, aluminum-doped zinc oxide (Al:ZnO) thin films were grown by high-speed atmospheric atomic layer deposition (AALD), and the effects of air annealing on film properties are investigated. The experimental results show that the thermal annealing can significantly reduce the amount of oxygen vacancies defects as evidenced by X-ray photoelectron spectroscopy spectra due to the in-diffusion of oxygen from air to the films. As shown by X-ray diffraction, the annealing repairs the crystalline structure and releases the stress. The absorption coefficient of the films increases with the annealing temperature due to the increased density. The annealing temperature reaching 600 °C leads to relatively significant changes in grain size and band gap. From the results of band gap and Hall-effect measurements, the annealing temperature lower than 600 °C reduces the oxygen vacancies defects acting as shallow donors, while it is suspected that the annealing temperature higher than 600 °C can further remove the oxygen defects introduced mid-gap states.

## 1. Introduction

Metal oxides are widely used in many applications such as optical coatings, transparent conducting oxides (TCOs), photocatalysis, sensors and photo-electrochemistry [1,2,3,4,5,6,7]. TCOs have attracted lots of attention because they show simultaneously conductivity and optical transparency. In the past fifty years, indium tin oxide (ITO) has become the most widely used TCO for optoelectronic devices due to its high transmittance of ~90% in the visible range and low resistivity (~2 × 10^−4^ Ω-cm) with mobility of about 40 cm^2^/Vs and high carrier concentration of 10^21^ cm^−3^ [8,9]. However, ITO faces several challenges. The scarcity of indium element may result in severely increased material cost. The commonly used sputtering process for preparing ITO can cause damage to the underlying layer as appears in the increased parasitic absorption at the TCO/Si interface of the heterojunction silicon solar cells [10,11,12]. The high carrier concentration of ITO reduces short-circuit current of solar cells if it is used as a window layer. The development of alternative materials with low cost and high mobility is necessary. Among all materials, aluminum-doped zinc oxide (Al:ZnO) is the most widely researched n-type TCOs owing to its excellent properties similar to ITO and comparability to various deposition techniques, such as sputter [13], spray pyrosis [14], plasma enhanced chemical vapor deposition [15], and pulsed laser deposition [16]. However, the Al:ZnO is generally used as an interconnection layer in optoelectronics, such as tandem thin film solar cells and polymer solar cells, but not used as an electrode since the ITO still outperform Al:ZnO in this aspect. Recently, atomic layer deposition (ALD) has become a very attractive approach to prepare TCOs [17,18]. The ALD technique is known for its unique features such as the deposition of conformal layer over a variety of substrate types and geometries, pinhole-free films with a very low defect concentration compared to other techniques, and the controllability of film thickness at sub-nanometer level. In some studies, ALD Al:ZnO has reached a low resistivity comparable to ITO [19]. It has been reported a better conversion efficiency of polymer solar cells with ALD Al:ZnO electrodes compared to that using ITO-coated glass [20]. Effects of the ALD deposition parameters such as substrate temperature, purge time, precursor dosing time on the properties of the Al:ZnO films are systematically studied [21,22,23,24]. However, the post-annealing effects on the ALD Al:ZnO are rarely reported. In the literature the post-annealing effects are mostly for magnetron sputtered Al:ZnO. For instance, Kim et al. reported an increased carrier concentration of the Al:ZnO films prepared by sputtering after vacuum annealing [25].Lin et al. reported a decreased carrier concentration in the case of nitrogen or oxygen annealing [26], while Zhou et al. observed a decreased resistivity of the Al:ZnO after annealing in nitrogen and oxygen mixture [27]. The results of the annealed Al:ZnO are controversial, and the related mechanism is not fully understand.

In this study, the Al:ZnO thin films were prepared using high-growth rate atmospheric ALD (AALD). The processing time of one ALD cycle is about 3 s, which is nearly 20 times shorter than vacuum-type ALD (~1 min for one ALD cycle). Furthermore, the growth rate per cycle of the AALD Al:ZnO films is 0.94 Å/cycle, corresponding to about 0.3 Å/s. This value is lower than that (0.5–3 Å/s) obtained by using sputter [28,29,30,31], but in terms of total deposition time, the AALD method would take more advantages since it does not require a vacuum environment. For example, for a 60 nm-thick Al:ZnO film, the AALD method takes about 40 min to finish the deposition, whereas the sputter technique may require more time as it needs to reach high degree of vacuum before deposition. The AALD process thus demonstrates high potential for the industrial applications. Instead of using traditional multilayered Al_2_O_3_/ZnO structure, the Al and Zn metal precursors are co-injected into the deposition zones. The films are annealed at 300–800 °C to manipulate the oxygen vacancies. The effects of the air annealing temperature on the optical, electrical and structural properties are investigated.

## 2. Results and Discussion

**Figure 1:**
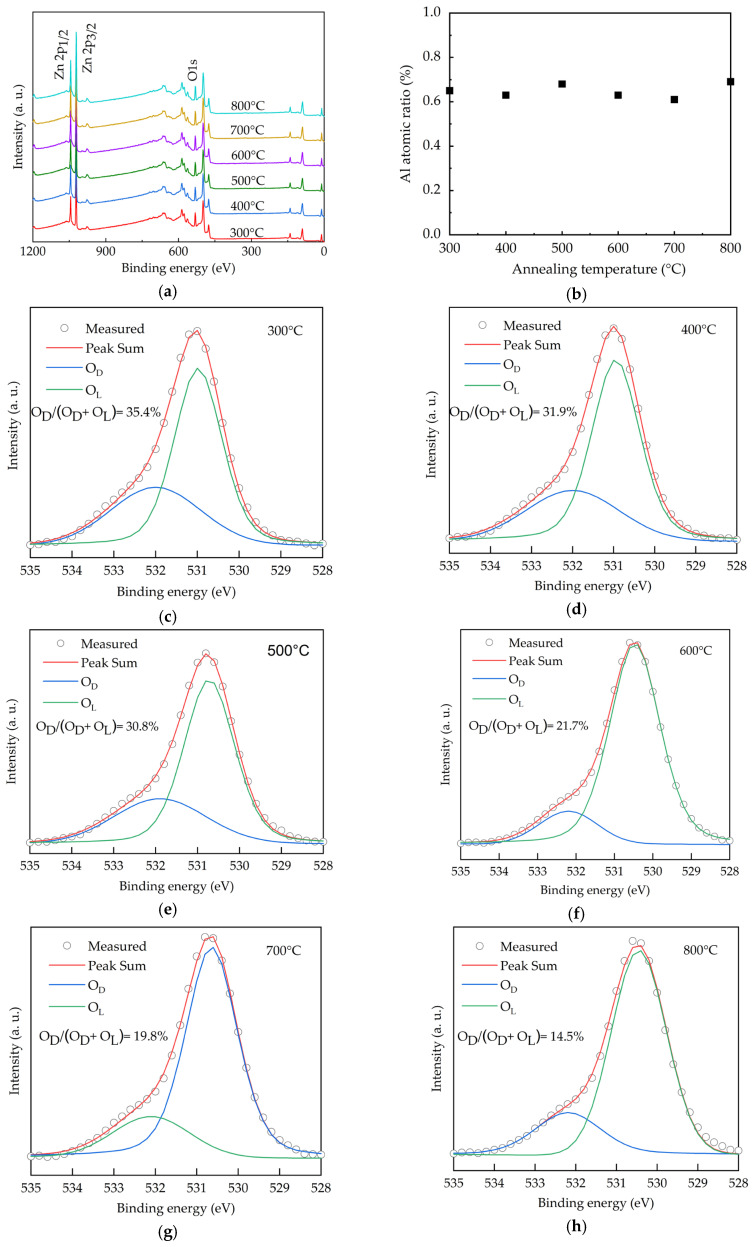
(**a**) XPS spectra, (**b**) Al content and (**c**–**h**) O 1s spectra for the AALD Al:ZnO thin films annealed at different temperature.

The chemical states of O for the AALD Al:ZnO thin films with different annealing temperatures are investigated by X-ray photoelectron spectroscopy (XPS), as shown in Figure 1a. The peaks at around 530, 1020, and 1050 eV are assigned to the O 1s, Zn 2p_3/2_, and Zn 2p_1/2_ core levels, respectively. No carbon peaks (~284.5 eV) are observable, indicating no or very low carbon content as the surface of the samples was sputtered before the XPS measurement to remove the surface contamination. In addition, the lack of carbon peaks suggests the nearly full ALD reactions of the diethylzinc (DEZ) and trimethylaluminum (TMA) precursors, otherwise the methyl groups in the precursors will remain in the deposited films. The Al peak at 75.6 eV is hardly observed due to the low content of <0.7 at.% as shown in Figure 1b. In order to investigate the chemical states, the O 1s spectra of the samples with different annealing temperature (Figure 1c–h) are deconvoluted into two peaks, which are centered at 530.6 and 532.1 eV. In the literature, a peak at the lower binding energies of 530–531 eV is generally attributed to the O^2−^ binding at lattice points of ZnO, while the peak at the higher binding energies (531–532 eV) is ascribed to oxygen vacancies [32,33,34,35,36,37,38]. We thus assign the peaks at 530.6 and 532.1 eV as the lattice oxygen (O_L_) and oxygen vacancies defects (O_D_), respectively. Increasing annealing temperature from 300 to 500 °C leads to high O_D_/(O_L_+O_D_) ratios of around 30.8%–35.4%. The ratio reduces to 27.4%, suggesting that the oxygen vacancies are filled by the oxygen from the annealing environment. As the oxygen vacancies affect optoelectronic properties of the Al:ZnO films [39], the significant variation of the O_D_/(O_L_ + O_D_) ratio from 35.4% to 14.5% indicates the high tunability of the film properties depending on the annealing temperature.

**Figure 2:**
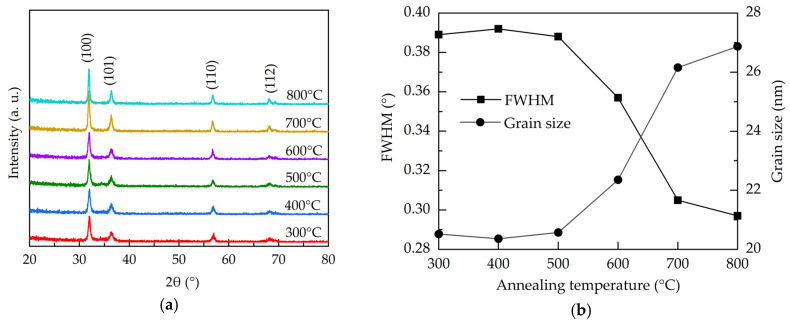

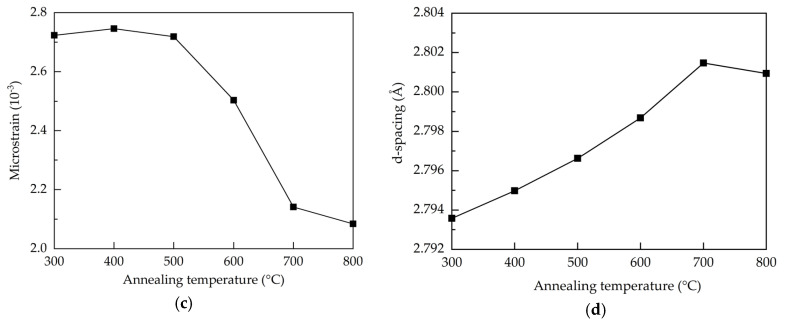
(**a**) XRD patterns, (**b**) FWHM and grain size, (**c**) microstrain, and (**d**) d-spacing of the AALD Al:ZnO thin films annealed at different temperature.

Figure 2a shows the X-ray diffraction (XRD) patterns for the atmospheric Al:ZnO films with different annealing temperature. All the films have the peaks at 31.83°, 36.36°, 56.69°, and 68.14°designated to (100), (101), (110), and (112) lattice planes of polycrystalline hexagonal wurtzite structure ZnO, respectively (JCPDS #36-1451) [40]. The preferred orientation is (100). All the samples do not show any peaks related to Al-related oxides, and this suggests that Al ions are well-incorporated into the wurtzite structure in the investigated annealing temperature range. The narrowing of the dominant (100) peaks with the increase of the annealing temperature indicates the improved crystallinity. Figure 2b illustrates the full-width-half-maximum (FWHM) of the (100) peaks and the corresponded crystallite size calculated using Scherrer′s equation as given by [41]:

(1) $$D\;=\frac{k\lambda }{\beta cos\theta },$$

where *D* is the average crystallite size, k is the Scherrer constant (0.9), *λ* is the wavelength of the X-ray radiation (0.15418 nm), *β* is FWHM, and *θ* is the Bragg’s diffraction angle of the (100) peak. At the annealing temperatures of 300–500 °C, the crystallite size is around 20.5 nm. Further increasing the annealing temperature from 500 to 800 °C leads to an enlargement of the crystallite size from 20.5 to 26.9 nm due to recrystallization of the films. In addition to the repair of the crystalline structure, the annealing treatment renders oxygen atoms/molecules able to diffuse from the air into the Al:ZnO films to fill the oxygen vacancies. The increased annealing temperature enhances this process, thereby improving the crystal growth and crystallite size. As oxygen vacancies are known to induce stress to the lattice structure [42], the reduction of the oxygen vacancies defects through high-temperature annealing releases the stress. This is also reflected on the decreased trend of the microstrain (ε), shown in Figure 2c, as calculated by [43]:

Ε = (0.9/D − 1/D)*λ/sinθ′(2)

Further, the restoration of the lattice structure should increase the interplanar distance between crystal faces. The interplanar distance (d-spacing) of the (100) preferred orientation for the samples is calculated by the following equation [44]:

2dsinθ = nλ,(3)

where n is the diffraction order. The 2-theta value shifts from 32° to 31.9° when the annealing temperature increases from 300 to 800 °C. Accordingly, the d-spacing increases from 2.79 to 2.801 Å as shown in Figure 2d.

**Figure 3:**
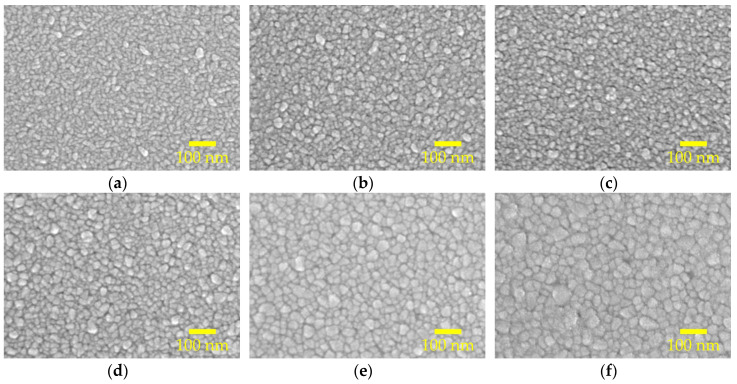
SEM morphological images of the AALD Al:ZnO films annealed at (**a**) 300, (**b**) 400, (**c**) 500, (**d**) 600, (**e**) 700, and (**f**) 800 °C.

Figure 3a–f show the scanning electron microscopy (SEM) topological images for the AALD Al:ZnO films with different annealing temperature. At 300 °C, the sample has elongated grains parallel to the substrate, which is in accordance with the XRD result showing a (100) preferred orientation. The grain size changes with varying the annealing temperature. By drawing a diagonal line, we can estimate the average grain size of the samples. The increasing trend of the average grain size with the annealing temperature agrees with the XRD result. In particular, the grain size well-matches the crystallite size determined from XRD at the annealing temperatures lower or equal to 600 °C, whereas at the annealing temperature of 800 °C the grain sizes are apparently larger than expected, suggesting that the high-temperature annealing causes the crystallites to aggregate rather than merge into larger crystals. The aggregation may lead to discontinuity/voids as clearly observed in Figure 3f. The presence of the voids can cause shunting or increased leakage current when the Al:ZnO films are used as the TCO of optoelectronic devices. Furthermore, in some applications the ALD films are mostly prepared at a thin thickness (few to tens nanometers), and the adverse influence of these voids could be more magnified. A proper annealing temperature is thus important for the AALD Al:ZnO films.

**Figure 4:**
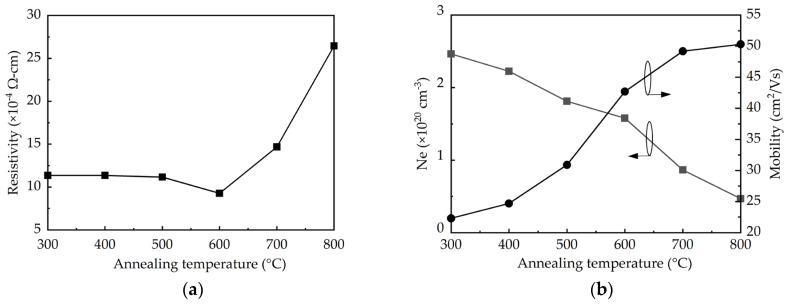
(**a**) Resistivity, (**b**) carrier concentration, Ne, and mobility determined by Hall-effect measurements as a function of annealing temperature.

The resistivity, mobility, and carrier concentration (N_e_) determined by Hall-effect measurements are shown in Figure 4. The resistivity remains 1.12 × 10^−3^ Ω-cm at the annealing temperatures of 300–500 °C, and then it decreases to a minimum of 9 × 10^−4^ Ω-cm at 600 °C, as shown in Figure 4a. Further increasing the annealing temperature to 800 °C leads to a significant increase in resistivity to 2.7 × 10^−3^ Ω-cm. It is known that the resistivity should be inversely proportional to the product of the mobility and carrier concentration. As shown in Figure 4b, the increased annealing temperature improves the mobility due to the enhanced crystalline structure and grain size. Noted that at 800 °C the relatively small increment of the mobility is possibly related to the adverse effect of the crystallite aggregation as discussed in the SEM results. In contrast, the carrier concentration reduces with increasing the annealing temperature as a consequence of the removal of the oxygen vacancies. Therefore, the net result of the competing effect of the increased mobility and reduced carrier concentration at increasing annealing temperature shows that the 600 °C-annealed sample has the lowest resistivity.

**Figure 5:**
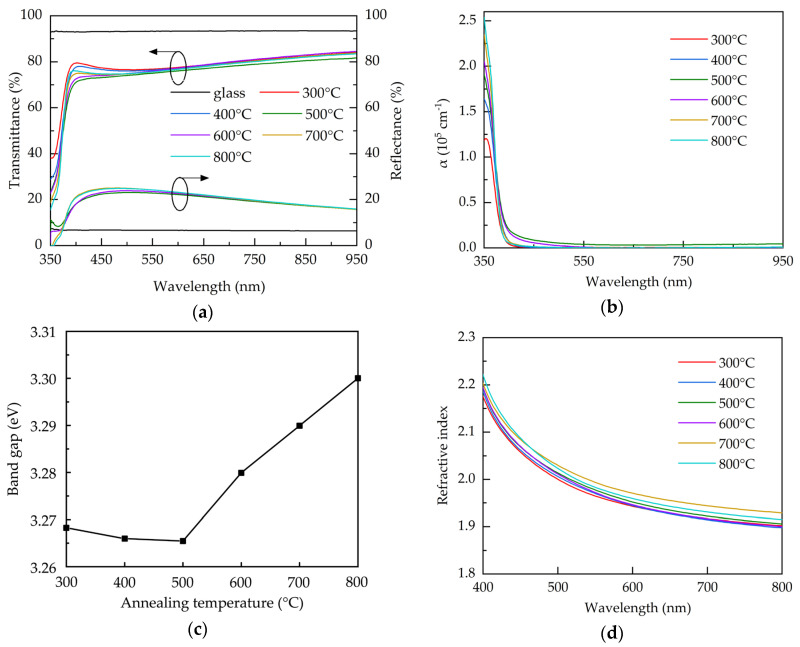
(**a**) Transmittance and reflectance spectra, (**b**) absorption coefficient spectra, (**c**) band gap, and (**d**) refractive index for the AALD Al:ZnO films with different annealing temperature.

The optical properties of the AALD Al:ZnO samples with different annealing temperature are investigated. Figure 5a shows the transmittance and reflectance spectra over the 350–950 nm wavelengths for the bare glass and the AALD Al:ZnO films annealed at different temperatures. Compared to the glass substrate, all the Al:ZnO films exhibit a lower transmittance, due to the significantly higher reflectance. To exclude the impact of the reflectance, the absorption coefficient (α) is used for assess the optical property of the films and calculated by:

(4) $$\alpha \;=\;-\frac{1}{d}\ln\left[\frac{T}{{\left(1-R\right)}^{2}}\right],$$

where *d* is the thickness of the films, *T* is the transmittance, and *R* is the reflectance. The result is shown in Figure 5b. The high absorption coefficients at the short-wavelengths (<400 nm) are due to the band-to-band absorption where the photon energy is close to the material band gap [45]. The increased absorption coefficient at increasing annealing temperature is related to the better crystalline structure with less oxygen vacancies. All the films exhibit similarly low absorption coefficients at the longer wavelengths. Figure 5c shows the band gap, calculated using Tauc’s plot method [46], of the AALD Al:ZnO films with different annealing temperature. The band gap slightly decreases from 300 to 500 °C. Further increasing the annealing temperature leads to a sharp increase in band gap. For Al:ZnO films, band gap variation is usually associated with the carrier concentration. When the carrier concentration exceeds the Mott critical density (~10^19^ cm^−3^ for ZnO-based films), the Burstein-Moss band filling effect occurs to increase the band gap and the increment is proportional to the N_e_^2/3^ [47,48]. This linear relationship is observed in the annealing temperatures of 300–500 °C in the present work. We therefore suspected that the reduction of the band gap can be attributed to the decreased carrier concentration and the less pronounced Burstein-Moss effect. The increased tendency of the band gap after 500 °C implies different mechanism. In addition to acting as the shallow donors, in some cases oxygen vacancies can introduce mid-gap states. The significant increase in band gap at 600–800 °C is possibly due to the reduction of the mid-gap states caused by the microstructure restoration or the removal of the mid-gap state oxygen vacancies. Similar results associated with the band gap enlargement due to the mid-gap state elimination in metal oxides are reported elsewhere [49]. Figure 5d shows the refractive indices of the samples annealed at different temperature. The refractive index increases with the annealing temperature increasing from 300–700 °C, due to the improved crystalline structure, as evidenced by XRD, and film density. The 800 °C-annealed sample has a reduced refractive index, which is commonly linked to the decreased density. As observed from SEM image, the enhanced grain boundaries and voids at 800 °C may account for the reduced density of the film.

## 3. Experimental

**Figure 6:**
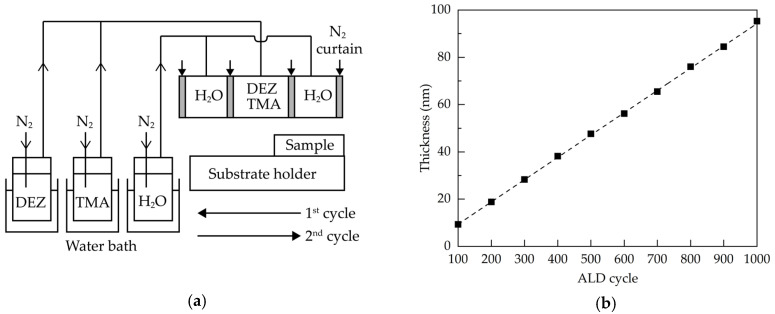
(**a**) Schematic diagram of the AALD system, and (**b**) thickness of the Al:ZnO films as a function of the ALD cycle.

Glass substrates with a size of 2 cm × 2 cm were ultrasonically cleaned in alcohol, acetone and deionized water for 15 min each, and then dried in nitrogen and placed in an oven at 80 °C for at least 30 min. The Al:ZnO films were deposited via a homemade AALD system having injector heads connected to trimethylaluminum (TMA), diethylzinc (DEZ) and oxidant (H_2_O). The metal precursors (purity: 99.9999%) were purchased from Aimou Yuan Scientific (Nanjing, China), and were co-introduced into the deposition region. The schematic diagram of the AALD system is shown in Figure 6a. The injection heads were arranged in the order of H_2_O, (DEZ+TMA), and H_2_O, each separated by nitrogen gas curtains. The temperatures of the TMA, DEZ, and H_2_O bubblers were controlled to be 40, 40 and 30 °C, respectively, using the heating water baths. Nitrogen gas was used as carrier gas, dilution gas and gas curtain. During AALD deposition, the samples were placed on a substrate holder in the air atmosphere. The substrate holder moved horizontally back and forth at a speed of 150 mm/s, and stopped for about 0.5 s when it reached both ends. The distance between the injection head and the substrate was 0.3 mm. Table 1 summarizes the detailed deposition parameters for the Al:ZnO films. 

When the substrate moved beneath the injector, the substrate firstly received the exposure of H_2_O to generate -OH species on the substrate surface. The substrate then moved to the metal precursors co-injection zone. The surface OH ligands reacted with the metal precursors to create O-Al or O-Zn bonds, and the surface species changed to -CH_3_ or -C_2_H_5_, as given by:

S-OH + Al-(CH_3_)_3_ or Zn(C_2_H_5_)_2_ → S-O-Al-(CH_3_)_2_ or S-O-Zn(C_2_H_5_) + CH_4_ or C_2_H_6_, (5)

where S denotes the substrate surface. Noted that there were exhaust holes between the H_2_O and the metal precursors injectors to remove the excess precursor molecules or by-products. When the substrate arrived the next H_2_O injection zone, the surface ligands changed to the initial state (-OH species) again as given by:

S-O-Al-(CH_3_)_2_ or S-O-Zn(C_2_H_5_) + H_2_O → S-O-Al-OH or S-O-Zn-OH + CH_4_ or C2H_6_ (6)

After the surface reactions (1) and (2), one ALD cycle was completed and repeated by moving the substrate back and forth beneath the injection head until the required film thickness was obtained. Figure 6b shows the film thickness as a function of the ALD cycle. The error of the film thickness was within ±1.5%. The linear relationship indicated the self-limiting nature of the ALD process. The growth per cycle was about 0.94 nm/cycle. To study the annealing effects on the properties of the films, the Al:ZnO films were prepared at a thickness of 60 nm, corresponding to the ALD cycle number of 635, and then received a post-deposition annealing process at different temperatures ranging from 300 to 800 °C in a diffusion furnace (FRD-002, Friend, Qingdao, China) in ambient air. The transmittance and reflectance spectra of the films were obtained by using a UV-visible spectrometer (MFS-630, Hong-Ming Technology, New Taipei City, Taiwan). The refractive indices and thickness of the films were determined by an ellipsometer (M2000, J. A. Woollam, Lincoln, NE, USA). The X-ray diffraction (XRD, Rigaku TTRAXIII, Ibaraki, Japan) measurements were carried out at an incident angle of 0.5° to investigate the crystal structure of the films. The X-ray photoelectron spectroscopy (XPS, ESCALAB 250Xi, Thermo Fisher, Waltham, MA, USA) with Al-Kα anode was employed to obtain the chemical states and elemental ratio of the films. The Hall-effect measurements (HMS5000, Side Semiconductor Technology, Shanghai, China) were conducted to determine the resistivity, carrier concentration, and mobility of the films. The surface morphologies of the samples were observed using a scanning electron microscopy (SEM, sigma 500, Zeiss, Oberkochen, Germany).

## 4. Conclusions

Al-doped ZnO films are prepared using high-growth rate AALD and receives a post-annealing process with different annealing temperature in air. The XPS result evidences the reduction of the oxygen vacancies, suggesting that the oxygen from the annealing ambient can be incorporated in the films to fill the vacancies. The XRD result also confirms the restoration of the crystalline structure. The annealing at 600 °C has the lowest resistivity of 9 × 10^−4^ Ω-cm due to the balance between the increased mobility and reduced carrier concentration at increasing annealing temperature. The band gap variation at 300–500 °C matches the Burstein-Moss effect, suggesting that the low annealing temperature mainly reduce the oxygen vacancies acting as donors. While the higher annealing temperature of above 600 °C could further reduce the mid-gap states. The AALD Al:ZnO thin films with high transparency and low resistivity provide a promising alternative option to replace the ITO films in solar cell applications.

## Figures and Tables

**Table 1:** Deposition parameters for AALD Al:ZnO films.

Parameter	Value
Annealing temperature (°C)	300–800
Substrate temperature (°C)	110
Substrate holder moving speed (cm/s)	15
Distance between injector and substrate (mm)	0.3
H_2_O carrier gas flow rate (sccm)	400
H_2_O dilution gas flow rate (sccm)	800
Precursor carrier gas flow rate (sccm)	200
Precursor dilution gas flow rate (sccm)	1100
TMA and DEZ bubbler temperature (°C)	40
